# Mucosal Immunity: The Key to Combating Enteroviruses and Beyond

**DOI:** 10.1155/jimr/4856137

**Published:** 2026-08-18

**Authors:** Nida Kalam, Vinod R. M. T. Balasubramaniam

**Affiliations:** ^1^ Jeffrey Cheah School of Medicine and Health Sciences, Monash University Malaysia, Jalan Lagoon Selatan, Bandar Sunway 47500, Selangor, Malaysia, monash.edu.my

**Keywords:** echovirus, enterovirus, enterovirus A71 (EV-A71), enterovirus D68 (EV-D68), gut-associated lymphoid tissue (GALT), human rhinovirus (HRV), innate immune evasion, mucosal immunity, mucosal vaccine, nasal-associated lymphoid tissue (NALT), oral poliovirus vaccine, oral rotavirus vaccine, poliovirus (PV), secretory IgA (SIgA), type I interferon (type I IFN), type III interferon (type-III IFN)

## Abstract

Enteroviruses (EVs) lead to 10–15 million symptomatic infections each year in the United States alone. EVs enter through either the gastrointestinal or respiratory mucosa, depending on the specific virus. Since these mucosal surfaces are the primary entry points for viruses, mucosal immunity serves as a crucial first line of defense. Nevertheless, the mechanisms that drive it are poorly understood for most EVs. Conventional intramuscular vaccines provide limited protection at these sites, leaving them unprotected; mucosal vaccination addresses this issue directly by generating local SIgA in GALT/NALT that neutralizes pathogens before they spread systemically, while also offering advantages in accessibility and cost. This is not merely theoretical; oral vaccines for polio and rotavirus have already shown the effectiveness of mucosal immunization at scale for enteric viral diseases. This review explores innate pattern recognition through TLR3, RIG‐I, and MDA5 pathways, type I/III interferon (IFN) signaling, and mucosal SIgA responses across key EVs. It also assesses current mucosal vaccine candidates for EV‐A71, CVA16, and EV‐D68, most of which remain in preclinical stages. Understanding of these mechanistic gaps is important for advancing successful mucosal vaccine strategies into effective next‐generation vaccines for EVs.

## 1. Introduction

Mucosal tissues in the gastrointestinal, respiratory, reproductive, and urinary tracts, as well as the eye surface, are key entry points for pathogens like viruses and serve as protective barriers. Mucosal immunity is vital for the body’s defense, involving responses at these surfaces through both innate and adaptive immune components, notably innate lymphoid cells and tissue‐resident memory T cells [1]. The innate immune system uses physical barriers like mucus and antimicrobial peptides for initial defense. If these barriers fail, dendritic cells (DCs) activate the adaptive immune response. Understanding this process is vital for developing vaccines and therapies to enhance localized immunity [2, 3]. Mucosal DCs capture antigens via M cells, thereby enabling the activation of adaptive immunity. At the effector site, plasma cells generate secretory immunoglobulin A (SIgA), which helps neutralize and eliminate antigens, thereby preventing infections at mucosal surfaces [4]. Oral poliovirus vaccine (OPV) stimulates the secretion of intestinal mucosal SIgA. This SIgA effectively neutralizes the virus at its main entry point, preventing replication and stopping person‐to‐person transmission through fecal–oral routes [5]. The mucosal barrier acts as a physical defense and includes mucosa‐associated lymphoid tissue (MALT), which is vital for immune responses to external antigens. About 50% of the body’s lymphocytes are found in MALT, including gut‐associated lymphoid tissue (GALT) and nasal‐associated lymphoid tissue (NALT) [1].

Enteroviruses (EVs) belong to the *Picornaviridae* family, cause 10–15 million symptomatic infections each year in the United States (US). They are small, nonenveloped viruses with a single‐stranded RNA genome [6]. Transmission mainly occurs through the fecal–oral route via contaminated surfaces, food, or water. While severe gastrointestinal symptoms are rare, complications can arise, allowing the viruses to spread to other tissues [6]. During viral infections, proteins and nucleic acids serve as pathogen‐associated molecular patterns (PAMPs), recognized by pattern recognition receptors (PRRs) like toll‐like receptors (TLRs) and RIG‐I‐like receptors (RLRs). These receptors detect viral nucleic acids, activating pathways that produce type I interferon (IFN) and cytokines [7, 8]. TLR2 recognizes human rhinovirus‐6 (HRV6) capsid [9], and TLR3 detects RNA from viruses like EV‐A71, triggering cytokine production [10]. Type I IFNs enhance T cell responses via the JAK‐STAT pathway, while type III IFNs manage infections at epithelial barriers [11, 12]. The mucosal immune system is crucial in halting EV replication, although its mechanisms are not well understood. This review emphasizes the importance of mucosal vaccines for EVs such as poliovirus (PV), enterovirus A71 (EV‐A71), coxsackievirus A16 (CVA16), human rhinovirus (HRV), coxsackievirus B (CVB), and enterovirus D68 (EV‐D68). It highlights successful examples, such as the OPV and oral rotavirus vaccine (ORV), and discusses current candidates in development. The review also addresses challenges and the urgent need for rapid vaccine development to prevent epidemics, while exploring antiviral immunity at mucosal sites related to EVs.

## 2. Searching Strategy

A narrative literature review was carried out utilizing PubMed/MEDLINE, Scopus, and Google Scholar, without restrictions on publication dates, as foundational research on mucosal immunity and PV vaccination is relevant to the newer EV literature. The search terms included “enterovirus,” “EV‐A71,” “CVA16,” “EV‐D68,” “human rhinovirus,” and “echovirus” with “mucosal immunity,” “innate immunity,” type‐I interferon,” type‐III interferon,” secretory IgA,” “GALT,” “NALT,” “dendritic cells,” “T cell response,” and “mucosal vaccine.” Additionally, reference lists from the retrieved articles were manually reviewed to identify further relevant studies. Articles were included if they discussed mucosal innate or adaptive immune mechanisms, immune evasion strategies, or the development of mucosal vaccines for the specific EVs. As this is a narrative rather than a systematic review, it did not follow a formal PRISMA screening or quality scoring protocol, a limitation mentioned in the Limitations section.

## 3. Mucosal Immunity and Advantages

Mucosal tissues in the gastrointestinal, respiratory, reproductive, and urinary tracts, as well as the eye surface, are vital entry points for pathogens, serving as protective barriers [1, 13]. Mucosal immunity is a vital part of the immune system, involving both innate and adaptive responses, notably including innate lymphoid cells and tissue‐resident memory T cells [1].

**Figure 1:**
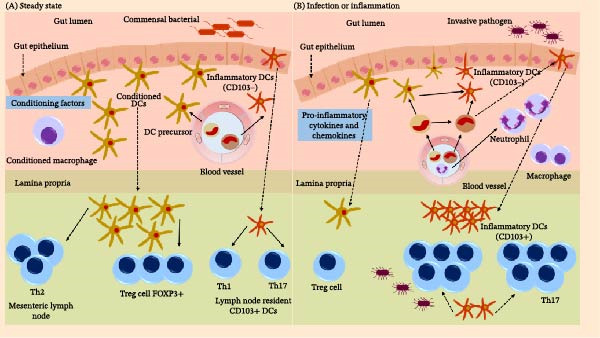
Panel A represents how dendritic cells (DCs) interact with both effector T cells and regulatory T (Treg) cells in response to commensal bacteria under normal conditions. In Panel B, the focus is on either anti‐inflammatory or proinflammatory reactions under normal conditions or during instances of infection or inflammation. Normally, such conditioning might also result from the sampling of commensal bacteria or exposure to self‐antigens or dietary proteins, leading to immune responses that are effectively “quieted” through tolerance mechanisms involving Treg cells or the action of IgA in immune exclusion. A smaller subset of another type of DCs might also migrate to the mesenteric lymph nodes under normal conditions. These cells, having possibly avoided prior conditioning, could push T helper cells toward Th1 or Th17 profiles. Although present in minor quantities, these cells do not cause disease under normal conditions but could serve as early warning systems upon encountering pathogenic bacteria/viral pathogens or in situations where abnormal reactions to soluble proteins lead to disease manifestations, such as in food allergies.

The mucosal immune system, referred to as MALT, encompasses a variety of anatomical locations, immune cells, and tissues. The functions of MALT can be categorized into three primary areas: (i) development of primary lymphoid tissues, (ii) initiation and enhancement of local immune responses, and (iii) generation of mechanisms for mucosal immunity [14]. The epithelial layer and lamina propria serve as the initial defense, while MALT triggers adaptive immune responses, especially involving T lymphocytes. In normal mice, there is one intraepithelial lymphocyte (IEL) for every 5–10 epithelial cells in the small intestine and one for every 40 in the colon [15]. Healthy adults have about 1 IEL for every 5–20 epithelial cells. Antigen‐presenting cells (APCs) capture antigens and migrate to lymphoid tissues, where T cells differentiate into effector cells. This IEL and lymphocyte network is crucial for mucosal barrier integrity and gastrointestinal health [16]. The LP acts as an effector site and is vital for B cell proliferation and maturation into plasma cells [17]. It also facilitates the T‐cell‐independent transition of B cells into IgA‐secreting cells in mice, though this remains debated due to inconsistent findings [18, 19]. The presentation of antigens by CD103+ DCs can lead to either tolerogenic or immunogenic responses, depending on the microenvironment [20, 21]. Under normal conditions, LP DCs exhibit tolerogenic behavior, but during inflammation, mesenteric lymph node CD103+ DCs transition to a proinflammatory state, stimulating Th1‐type responses and increasing IL‐6 production, without changes in phenotype or development [21, 22], as shown in Figure 1.

GALT includes organized regions for lymphocyte activity, as well as dispersed areas for activated cells [23]. Goblet cells produce mucus to strengthen the mucosal barrier, which contains defensins and host defense proteins [24]. Immune responses to enteric viruses occur primarily in the intestinal mucosa, involving the epithelium and lamina propria. Reactions to gut pathogens begin in lymph nodes and GALT, such as Peyer’s patches [25]. GALT is essential for antigen sampling and adaptive immune responses in the intestinal wall. In humans, GALT consists of Peyer’s patches, the appendix, and isolated lymphoid follicles [26]. Bovine studies indicate comparable dendritic and T cell levels in neonatal and post‐weaning calves, suggesting a functional mucosal immune system at birth. DCs in GALT facilitate T cell differentiation between dietary antigens, commensal microbiota, and pathogens, thereby enhancing specific immune responses [27].

## 4. DCs Induced Mucosal Immunity

An alternative method for developing a dendritic cell (DC) targeted vaccine involves using specific adjuvants like DC growth factors or TLR ligands found on mucosal DCs. Intranasal administration of plasmids that encode for Fms‐like tyrosine kinase 3 (Flt3) ligand and CpG oligonucleotide leads to the selective targeting of CD8α+ DCs and pDCs, respectively [28, 29]. CD8α+ DCs facilitate cross‐presentation and the induction of Th1 and cytotoxic T lymphocyte (CTL) responses, whereas pDCs are major producers of type I interferons (type I IFNs) rather than Th1 cytokines per se, thereby driving antigen‐specific IgA antibody responses and cellular immunity. Research indicates that applying soluble antigen alongside cholera toxin (CT) directly to the skin engages MLN langerin+ DCs in fostering intestinal IgA production through an RA‐dependent pathway [30]. Similarly, systemic vaccination using flagellin enhances the recruitment of CD103+ DCs to MLNs and boosts intestinal IgA antibody responses [31]. Additionally, the intra‐tracheal delivery of LPS‐treated, immunodominant CTL epitope‐loaded DCs represents a promising approach to creating CTLs capable of safeguarding against respiratory infections by intracellular microbes [32].

## 5. Secretory IgA (SIgA): Role in Mucosal Immunity

Secretory IgA (SIgA) is a vital component of the mucosal immune system, with production in healthy humans averaging 60 mg per kilogram of body weight daily, primarily from the gastrointestinal tract (GIT); this is double the daily output of IgG [33]. T lymphocytes (CD4+ and CD8+) predominantly comprise the mucosal effector cells, accounting for ~80% of mucosal lymphoid cells, as shown in Figure 1. SIgA serves as a primary defense mechanism against enteric pathogens and toxins that breach the intestinal epithelium. Its production is regulated by key cytokines, including interleukins (IL‐5, IL‐6, and IL‐10) and transforming growth factor‐β (TGF‐β), which play a crucial role in maintaining mucosal tolerance and establishing the interplay between SIgA production, immune response, and intestinal homeostasis [34].

**Figure 2:**
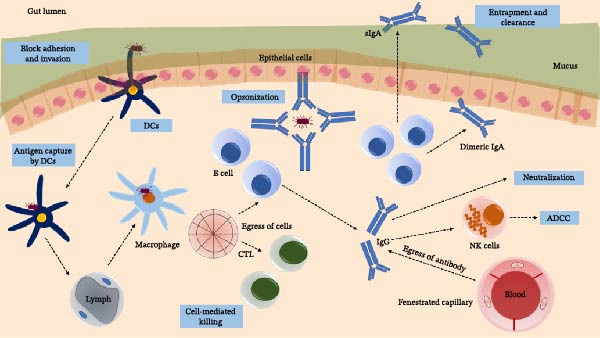
At mucosal surfaces, protection is afforded through various immune responses. Effector B and T cells that are antigen‐specific circulate in the bloodstream and identify mucosal high endothelial venules (HEVs) to migrate into the mucosal tissue. B cells undergo final differentiation into plasma cells, predominantly generating dimeric IgA. This IgA is then secreted as SIgA into the mucosal secretions, where it plays a vital role in intercepting pathogens and antigens, thus preventing their invasion of the mucosa. Additionally, neutralizing IgG antibodies are found within the mucosal tissues, which may originate from either local plasma cells or be diffused from the blood through the fenestrated capillaries in the vicinity. Specific cytotoxic T lymphocytes (CTLs) or the process of antibody‐dependent cell‐mediated cytotoxicity (ADCC), which involves a partnership between natural killer (NK) cells and antibodies, can eliminate infected cells. Moreover, dendritic cells (DCs) and macrophages can capture pathogens and transport them to the draining lymph nodes for further immune response.

Zhaori et al. [35] reported that oral polio vaccine (OPV) induced a high SIgA response, in contrast to inactivated polio vaccine (IPV). In a preclinical porcine model, a live attenuated trivalent rotavirus vaccine showed promising results in piglets. It protected against rotavirus‐induced diarrhea and prevented lesions in the small intestine when piglets were challenged with similar strains. Additionally, the vaccine triggered the detection of SIgA in fecal samples and its secretion at the intestinal lumen in immunized piglets. These preclinical findings warrant further validation in human studies [36]. Altogether, mucosal immunity is crucial in eliminating invasive pathogens, specifically enteric pathogens. SIgA is one of the dominant arms of mucosal immunity; it enables and primes itself in the presence of the enteric pathogen and helps eliminate pathogens [34]. Besides its antagonistic potential against toxins, pathogenic bacteria, and viruses, SIgA is integral in maintaining mucosal homeostasis. It aids in the retrotransport of antigens throughout the intestinal epithelium towards subsets of DC present in GALTs and NALT; additionally, it modulates intestinal microbiota composition and suppresses proinflammatory activity evoked by pathogen or pathogen allergenic antigens [34], as shown in Figure 2.

## 6. EV‐Specific Mucosal IFN Response

### 6.1. Induction of IFNs at Mucosal Sites in EV Infection

The innate immune system serves as the primary defense against pathogens and plays a key role in initiating adaptive immune responses. It detects invaders through pattern‐recognition receptors (PRRs), which identify conserved elements known as PAMPs. Major PRR families in mammalian innate immunity include TLRs, NOD‐like receptors (NLRs), C‐type lectin receptors, RLRs, and cytosolic DNA sensors like IFI16, DDX41, and cGAS [37, 38]. These cytosolic components of the innate immune system play a crucial role in transmitting signals from PRRs to downstream pathways like IRF3/7, NF‐κB, and MAPK. This signaling activates inflammatory cytokines and type I interferons (IFNs), vital for initiating innate immune responses [39].

Type I IFNs are essential against viral pathogens, functioning to activate interferon‐stimulated genes (ISGs) through JAK‐STAT pathways, establishing an antiviral state in infected and nearby cells. They also help mature DCs for better antigen presentation to T cells, promoting targeted adaptive immune responses [40, 41]. PRRs such as TLRs, RIG‐I, and MDA5 detect dsRNA of EV‐A71, activating signaling pathways that phosphorylate IRF‐3 and IRF‐7 and activate NF‐κB. This leads to IFN production, which binds to cell surface receptors, causing expression of ISGs and antiviral proteins. IFNs also enhance immune responses by promoting MHC class I expression, increasing co‐stimulatory molecules on DCs, supporting T cell proliferation, and enhancing adaptive immunity [42, 43].

TLR3 levels are elevated in human intestinal epithelial cells (IECs) during EV infections. The activation of IRF1, an IFN regulatory factor, enhances antiviral defenses, particularly in the respiratory system. A study revealed a mechanism for type III IFN production via the TLR3/IRF1 pathway in the intestine following EV infections, representing a regulatory strategy for antiviral defense [44]. Research has shown that EV‐A71 evaded detection and destruction by IECs, intraepithelial lymphocytes (iIELs), and innate natural killer (iNK) cells. It was identified that EV‐A71 infection led to increased production of type III IFN (IFN‐λ) compared to type I IFN (IFN‐β) in IECs. While IFN‐λ limited EV‐A71 replication, the virus significantly reduced the antiviral responses triggered by this IFN [45].

### 6.2. Induction of Innate Immunity at Mucosal Sites: During EV Infection

#### 6.2.1. PV

Poliomyelitis is a viral disease caused by PV belonging to the *Picornaviridae* family, primarily affecting children under five. There are three serotypes: PV1, PV2, and PV3, with limited cross‐immunity. The virus spreads via the fecal–oral route. About 75% of infections are asymptomatic, while 24% show mild symptoms, and around 1% lead to acute flaccid paralysis (AFP). Global efforts have reduced cases by 99% over 40 years, with wild PV types 2 and 3 declared eradicated in 2015 and 2019, respectively. Currently, wild poliovirus type 1 (WPV1) is limited to Afghanistan and Pakistan, with vaccine‐derived cases still emerging in Asia and Africa [46].

PV is a key EV for evaluating mucosal vaccine efficacy but is often overlooked in mucosal immunology. It infects through the oral route, replicating in the oropharynx and intestines before entering GALT. Unlike EV‐A71 and CVA16, PV uses the poliovirus receptor (PVR/CD155), found on intestinal M cells and DCs. The expression of CD155 affects susceptibility; humans and some primates express it, while rhesus macaques and CD155‐transgenic mice have reduced levels, leading to resistance. This specificity makes these models ideal for studying PV pathogenesis [47]. PV shares an intestinal defense pathway with EV‐A71 and CVB3, specifically through TLR3/IRF1‐driven production of type III IFN (IFN‐λ), which limits PV replication in IECs. This positions PV among other EVs rather than as an anomaly. PV requires the TLR3 adaptor TICAM‐1 (TRIF) for innate immune sensing; mice lacking TICAM‐1 have a severely diminished cytokine response to PV and increased mortality [48].

#### 6.2.2. EV‐A71

EV‐A71 is known to cause significant outbreaks and hospitalizations, particularly in the Asia‐Pacific region, with estimated annual hospitalizations ranging from 100 to 1000. Infection can lead to serious neurological conditions, including brainstem encephalitis, AFP, and aseptic meningitis, along with complications such as pulmonary edema and neurogenic pulmonary hemorrhage [49]. Currently, effective treatments and vaccines for EV‐A71 are lacking. In China, three formalin‐inactivated vaccines have been approved, while an inactivated vaccine, EnVax‐A71, in Taiwan has completed phase III trials, showing 99.2% efficacy (95% CI: 94.3–99.9, *p* < 0.001) [50]. Human intravenous immunoglobulin (IVIG) is employed for managing EV‐A71‐related brainstem encephalitis, although research has noted an antibody‐dependent enhancement phenomenon in these patients [12]. As PV faces imminent eradication, neurotropic NPEVs such as EV‐A71 may emerge as significant sources of morbidity and mortality [51].

**Figure 3:**
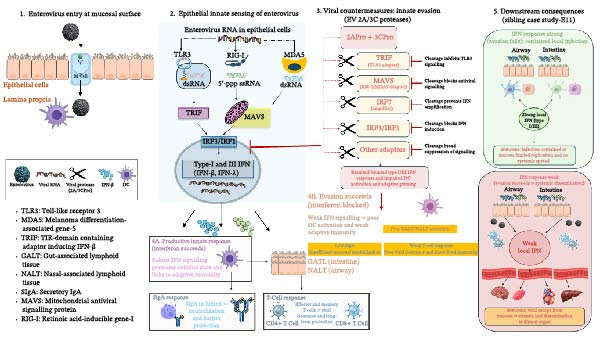
Mucosal immune response to enterovirus infection and enterovirus evasion mechanisms. This diagram depicts the host immune response at mucosal surfaces and the strategies used by the enteroviruses to counteract it. (1) Viral entry: Enteroviruses, like PV, enter through M cells and infect epithelial cells. (2) Innate immune detection: Viral RNA is identified by PRRs such as TLR3 and RIG‐I, which signal through TRIF and MAVS, activating IRF3 and IRF1 to produce type I and III interferons (IFN‐I/III). (3) Immune response: Interferon signaling promotes dendritic cell maturation and migration to lymphoid tissues, initiating adaptive immunity, resulting in SIgA production and T‐cell activation. (4) Viral evasion: Enteroviral proteases 2A and 3C impede antiviral signaling by cleaving crucial molecules like TRIF and MAVS, reducing interferon production and immune responses. (5) Clinical outcome: Effective interferon responses limit viral replication to mucosal tissues, while impaired signaling allows for viral spread, resulting in viremia. The inset contrasts local containment with widespread dissemination in cases of impaired responses, with red pathways indicating viral antagonism and blue/green pathways showing protective immune responses.

EV‐A71 spreads via the oral–fecal route with viral particles identified in feces that infect others [52]. Research indicates that differentiated IECs can be targets for certain picornaviruses. Studies show that colorectal cancer‐derived HT‐29 cells are susceptible to EV‐A71 infection, implying that IECs may act as initial targets for viral replication and shedding during the early stages of infection [45, 53]. EV‐A71 is transmitted through the intestinal tract, yet its mechanisms for evading the intestinal mucosal immune system are not well understood. However, research indicates that EV‐A71 infection elevates type III IFN (IFN‐λ) levels more than type I IFN (IFN‐β) in IECs, with IFN‐λ effectively limiting EV‐A71 replication. The viral proteases 2A (2Apro) and 3C (3Cpro) inhibit IFN‐λ production and receptor expression, thus reducing the IEC response to IFN‐λ. Additionally, EV‐A71‐infected IECs exhibit decreased susceptibility to lysis by intestinal NK (iNK) cells and CD3+ intraepithelial lymphocytes (iIELs). The viral proteases also downregulate NKG2D ligands and increase PD‐L1 expression on IECs, facilitating immune evasion [45]. At the intestinal mucosal surface, the TLR3/IRF1/type III IFN pathway is the most well‐documented innate defense mechanism. Su et al. [44] showed that the TLR3‐mediated activation of IRF1 in IECs stimulates the production of type III IFN (IFN‐λ) and limits EV replication in the gut, as shown in Figure 3. This pathway serves as the most clearly established innate immune response for EV‐A71 at the mucosal surface. The subsequent innate signaling facilitates the maturation of DCs and activates the adaptive immune response in the GALT [40, 41]. Research indicated that the mucosal vaccines shown that administering the EV‐A71 antigen orally and intranasally stimulates the production of VP1‐specific SIgA in intestinal, vaginal, and respiratory secretions in animal models. This method provides a significant protection that parenteral vaccination does not achieve, supporting the preference for mucosal routes in EV‐A71 vaccine delivery [54–56].

#### 6.2.3. CVA16

CVA16 is known to cause complications such as encephalitis, myocarditis, and pneumonitis in some patients, potentially leading to fatal outcomes [57]. CVA16 spreads via the fecal–oral route and infects the gastrointestinal epithelium, triggering mucosal immune responses through GALT [58–60]. CVA16 is recognized as a significant pathogen, but its innate immune evasion mechanisms at mucosal surfaces are less understood than EV‐A71. 3Cpro of multiple EVs inhibits MDA5 and disrupts type I IFN signaling through MAVS. Additionally, 3Cpro cleaves TAK1, blocking NF‐κB activation and dampening TLR‐mediated antiviral responses. Evidence also suggests a unique mucosal defense mechanism involving intestinal tuft cells [61]. Chen et al. [62] demonstrated that CVA16 infection expands intestinal tuft cells via the IL‐25/tuft cell pathway. Tuft cells trained by IL‐25 are more responsive to subsequent viral infections due to SAT1‐mediated depletion of intracellular polyamines, which inhibit enteroviral replication [62]. These findings highlight an important aspect of mucosal innate immunity against CVA16 and emphasize the need for further mechanistic studies beyond those offered by humoral vaccines. Nasal immunization with a CVA16‐VLP vaccine adjuvanted with CpG effectively stimulated IgA production in the respiratory tract and other mucosal sites, such as the intestines and vagina. This study showed that CpG enhanced nasal delivery of CVA16‐VLPs, leading to significant mucosal and systemic immune responses, including high levels of neutralizing antibodies and SIgA secretion in various mucosa. Overall, CVA16‐VLP immunization via the mucosal route elicited both cellular and humoral immune responses in mice [63]. This study represents the importance of mucosal immunization for the treatment of EVs.

#### 6.2.4. EV‐D68

Unlike other EVs, EV‐D68 primarily causes respiratory illnesses but has also been associated with neurological disorders like AFP, particularly during the 2014 outbreak [64, 65]. Research has found that type I IFN (IFN‐β) responses to EV‐D68 occur after type III IFN. This study also noted significant IFN production despite undetectable viral load, indicating a strong response from airway epithelial cells [66]. Research has identified innate immune sensing mechanisms that shape the respiratory mucosal response to EV‐D68. PRRs such as TLR3, TLR7, RIG‐I, and MDA5 detect viral RNA on airway epithelial cells, triggering MyD88/TRIF and MAVS signaling pathways [67]. However, EV‐D68 counters these responses; 3Cpro of EV‐D8 cleaves TRIF, disrupts RIG‐I signaling, and cleaves IRF7, while the 2C protein inhibits NF‐κB activation, reducing type I IFN production. Additionally, the structural protein VP3 interacts with IRF7 to inhibit its phosphorylation and nuclear translocation, offering an alternative immune evasion strategy [68]. MDA5‐mediated type III IFN induction has also been noted as a key detection pathway in infected airway cells [69]. These findings highlight EV‐D68s multifaceted strategies to evade immune responses, but further research is necessary to assess the significance of each pathway in primary airway epithelial cells in vivo.

EV‐D68 targets respiratory tissues, with NALT being essential for mucosal adaptive immunity. Thus, intranasal vaccination effectively promotes protective SIgA in nasal and bronchial secretions [55]. Lin et al. [55] supports this, demonstrating that intranasal immunization with formalin‐inactivated EV‐D68, along with polysaccharides from *Ganoderma lucidum* (PS‐G), triggered the production of EV‐D68‐specific IgG and SIgA in the respiratory secretions and various mucosal compartments of immunized mice. This approach yielded higher levels of neutralizing antibodies compared to a vaccine that used CpG as an adjuvant [55]. However, the specific innate immune signaling pathways activated by EV‐D68 at the respiratory mucosal surface remain incompletely understood, warranting further investigation.

#### 6.2.5. CVB

Group B coxsackieviruses (CVB1‐6) are important EVs associated with myocarditis, aseptic meningitis, pancreatitis, neonatal diseases, and potential triggers of type 1 diabetes. They enter the body through the fecal–oral route, infecting the intestinal epithelium. CVB uses two main receptors: the coxsackievirus and adenovirus receptor (CAR) and decay‐accelerating factor (DAF/CD55) for cell entry. Similar to PV, CVB crosses the intestinal barrier via M cells in Peyer’s patches, replicating in lymphocytes and macrophages before spreading throughout the body [70, 71]. CVB suppresses the intestinal IFN response during primary replication. In human intestinal epithelial cell models (Caco‐2 and HT‐29), CVB3 infection did not induce the typical strong types I and III IFN responses seen with viral RNA mimics. This reduced response is linked to the virus‐induced depletion of crucial proteins like TRIF, MAVS, and eIF4G, essential for IFN activation. This indicates that CVB disrupts innate signaling in intestinal cells rather than merely evading it. This aligns with evasion tactics in EV‐A71 and EV‐D68, showing type I/III IFN suppression as a shared target among different EVs [72]. Mucosal vaccine development for CVB lags behind that of EV‐A71 and parenteral vaccines against CVB, highlighting the need for more research in this area. A formalin‐inactivated CVB1 vaccine showed promise in preclinical studies, leading to PRV‐101, which recently completed a Phase I trial with good tolerability and antibody responses [73]. However, all formulations have been parenteral, neglecting the critical evaluation of oral or intranasal options for CVB’s intestinal immune response.

#### 6.2.6. HRV

HRV, commonly known as the *"*common cold virus,*"* has traditionally been overlooked as a significant contributor to severe illnesses [74]. HRV primarily infects and replicates in epithelial cells. Studies using human airway and primary bronchial epithelial cells have highlighted the inflammatory and innate immune responses to HRV. Epithelial cells predominantly produce type III IFN (IFN‐λ1) rather than type I IFN in response to HRV infection [75, 76]. Warner et al. [77] found that highly differentiated epithelial cells have higher levels of IFN‐λ1 mRNA and protein compared to IFN‐β. Human mucosal samples highlight the importance of early mucosal defenses in respiratory infections [66, 78]. Using an organotypic model of nasal epithelium, research showed that innate immune signaling pathways affect the inflammatory response to HRV, primarily activating an IFN response in healthy tissue, which restricts infection to <2% of cells. Inhibiting this response increased viral replication and proinflammatory activity via NF‐κB and NLRP1, along with increased mucus production from IL‐1β release. These findings reveal crucial host responses to HRV and suggest potential therapeutic targets [79].

The humoral and innate mucosal responses to HRV are well‐studied, but T cell‐mediated immunity in the respiratory mucosa is less examined. CD4+ and CD8+ memory T cells recognizing HRV antigens are present in peripheral blood. HRV stimulates DC maturation, increasing MHC class II, CD80, and CD86 expression, which enhances antigen presentation to T helper (Th) and cytotoxic lymphocytes [80]. In individuals with asthma, HRV infections may shift Th responses to a Th2 profile due to alarmin release, potentially worsening mucosal pathology [81]. There is a significant research gap regarding tissue‐resident memory T cells (Trm) in the nasal mucosa and NALT after HRV infection, crucial for respiratory antiviral immunity. Addressing this gap is essential for developing cross‐protective mucosal vaccines against diverse serotypes of HRV.

IgA has been demonstrated to be highly effective in addressing viruses within infected epithelial cells and in redirecting antigens to the lumen upon their entry into the lamina propria [82]. Upper respiratory infections associated with viruses, such as HRV, have been shown to induce an increase in levels of SIgA in nasal lavage samples [83]. The significant antigenic diversity of HRV, which includes more than 160 distinct serotypes, represents a considerable barrier to the development of a broadly effective mucosal vaccine. Ongoing research into the HRV‐specific innate immune mechanisms in the nasal mucosal epithelium, as well as the adaptive immune responses in NALT, is currently insufficient. Additionally, this line of research does not reflect the same level of specificity achieved with PV and EV‐A71 [74].

### 6.3. Echovirus

Human echoviruses belong to the EV B species within the Picornaviridae family. They are now known to cause various illnesses, especially in neonates, children, and immuno‐compromised individuals. These nonenveloped, positive‐sense single‐stranded RNA viruses transmit mainly via the fecal–oral route but also through respiratory droplets and vertical transmission. They replicate in the oropharyngeal and intestinal mucosa, then spread to target organs such as the central nervous system, heart, and liver. Clinical symptoms can range from mild febrile illnesses and rashes to severe conditions such as aseptic meningitis, neonatal sepsis‐like syndrome, encephalitis, and myocarditis [84]. E11 frequently causes pediatric infections with seasonal patterns and generally self‐limiting courses [85]. A clinical study found that male nonhomozygotic twins are at higher risk for severe complications from a new E11 variant linked to massive liver failure. Although the reasons for this increased pathogenicity are unclear, differences in immune responses at the mucosal level may contribute. EVs typically target the gastrointestinal or respiratory tracts before affecting other organs. In the study, a newborn without complications had a strong local IFN response, while the sibling with a fatal outcome had a strong airway antiviral response but a weak gastrointestinal response and high viral load, suggesting that poor control in the intestines may allow E11 to spread systemically [86]. There is a gap in understanding innate immune responses during E11 infection in the intestinal mucosa. It is unclear whether the reduced IFN response in severe cases is due to viral evasion, immature neonatal immunity, or genetic factors. This highlights the need for further research, especially since there are no approved antiviral treatments for neonates.

### 6.4. FDA‐Approved Mucosal Vaccines Against Enteric Pathogens

#### 6.4.1. OPV

PV is part of the EV genus within the Picornaviridae family. Live attenuated PV vaccines have been essential for vaccination efforts and eradicating wild‐type viruses. OPV extensively replicates in the GI tract, promoting intestinal immunity and reducing virus shedding upon re‐exposure [87]. Conversely, the inactivated poliovirus vaccine (IPV) offers strong systemic immunity and prevents paralytic disease but does not significantly reduce intestinal viral replication after exposure to the OPV in individuals unexposed to the virus [88]. Two vaccines have been approved for PV prevention: IPV in 1955 and the trivalent OPV in 1963, which replaced IPV. OPV generated local immunity in the intestines and antibodies against all three polio strains [89]. However, OPV‐2 strains can revert to a virulent form, leading to outbreaks of vaccine‐derived PV. Consequently, the vaccination program now includes bivalent OPV (excluding strain‐2) and a dose of trivalent IPV. Although IPV cannot cause circulating vaccine‐derived polioviruses (cVDPVs), it induces weaker intestinal immune responses than OPV, especially in OPV‐naive individuals [90].

In 1988, the Global Polio Eradication Initiative (GPEI) introduced the OPV as the main tool for polio eradication due to its cost‐effectiveness and reliable supply [91]. The global incidence of polio significantly declined from 350,000 cases in the 1980s to just 22 in 2017, marking a reduction of over 99.99% [92]. Recently, the OPV2 was tested in phase II trials, with results showing safety and no genetic reversion [93]. Interestingly, a study found that individuals shedding the virus had higher initial IgA levels after OPV vaccination compared to nonshedders, indicating that the vaccine induces a stronger mucosal immune response in some individuals [94]. Furthermore, research highlighted that early childhood immunization plays a crucial role in developing long‐lasting immune responses and promoting IgA production, which helps limit PV replication in the gut. However, age‐related decreases in mucosal IgA and prolonged virus shedding raise important considerations for polio eradication strategies [95].

#### 6.4.2. ORV

Rotavirus is not an EV, yet its oral vaccines illustrate that mucosal vaccination can confer population‐level protection, serving as a benchmark for EV‐specific candidates [96]. Rotaviruses are responsible for causing enteric infections such as severe diarrhea and diarrhea‐related deaths in children <5 years of age [97]. Oral and intravenous rehydration is the first‐line treatment for children; rotavirus infection can be life‐threatening or fatal for the population living with limited healthcare infrastructure. Approximately 150,000–200,000 deaths occur yearly [98]. In 2006, WHO approved Rotarix and RotaTeq for infants and children [99]. Both vaccines have a small, documented risk of intussusception (about 1–6 additional cases per 100,000 vaccinated infants), which is far outweighed by their benefits in preventing rotavirus mortality and hospitalizations. This risk is monitored through post‐marketing surveillance and included in vaccination guidelines [100]. A meta‐analysis reviewed studies on the impact and effectiveness of approved rotavirus vaccines in 32 countries across the globe. The meta‐analysis reported that the standard dosing schedules of Rotarix (2‐dose series) and RotaTeq (3‐dose series) decreased the mortalities among children and prevented hospitalizations for rotavirus‐induced diarrhea [101]. A database‐based analysis from 2001 to 2016 demonstrated that the rotavirus vaccine prevented hospitalizations due to rota‐induced gastroenteritis (RVGE) and all‐cause acute gastroenteritis (AGE) among children aged 0–4 years [102]. Middleton et al. [103] systematically analyzed the effect of the booster oral rota vaccine on immunogenicity and reduction in the incidence of gastroenteritis. It was found that ORVs exhibit reduced efficacy in high‐mortality regions. Findings indicate that an extra dose enhances IgA immune response significantly: 74.3% for the booster dose vs. 56.1% for the standard regimen, especially in seronegative children at baseline [103]. Only one study showed a slight reduction in gastroenteritis in the first 2 years of life. Although booster doses can improve immunogenicity, their effect on lowering clinical disease incidence is not consistently evident [103].

### 6.5. Mucosal Vaccines for EVs: Under Development or in the Preclinical Phase

Mucosal vaccination induces an immune response by delivering antigens through mucosal routes, where M cells take up the antigen and APCs process it. This activates B‐cells, resulting in IgA production influenced by Th2 cytokines [104]. The GIT is ideal for enteroviral entry, enhancing IgA secretion for protection. However, safe adjuvants for human mucosal vaccines are still unavailable [104–106].

A recent study developed an EV‐A71‐killed vaccine using nanosized calcium phosphate adjuvant encapsulated in alginate and chitosan carriers. The chitosan‐based vaccine produced a robust immune response, with high levels of IgA secreted upon oral delivery, unlike the alginate version. The study also highlighted the significance of dual delivery systems (intradermal and oral) for inducing both mucosal and systemic immune responses [107]. Similarly, the rVP1 EV‐A71 vaccine developed with chitosan enhanced VP1‐specific IgA antibodies in various tissues of ICR mice. Vaccinated mice had higher levels of IFN‐γ, IL‐4, and TGF‐β. Remarkably, 30% of newborns from immunized mothers were protected from a lethal dose of EV‐A71 [56]. Subsequently, a study assessed two synthetic lipopeptides, Pam3CSK4 and fibroblast‐stimulating lipopeptide‐1 (FSL‐1), which interact with TLR2/1 and TLR2/6, respectively, as adjuvants for a mucosal vaccine. Animal studies indicated that mice immunized with EV‐A71+FSL‐1 exhibited higher IgG and IgA levels than those receiving EV‐A71+Pam3CSK4 or a control group. Additionally, EV‐A71+FSL‐1 elicited a stronger T‐cell response and increased IFN‐γ and IL‐17 levels, along with strong neutralization against the EV‐A71 C2 subgenotype and cross‐neutralization of B4 and B5 subgenotypes [108].

CpG oligodeoxynucleotides (ODNs) linked to the EV‐A71 vaccine were tested in mice to boost immune responses. By mimicking bacterial DNA, CpG ODNs activate TLR‐9 on B cells and DCs, leading to enhanced Th1 responses. The CpG‐ODN‐EV‐A71 vaccine produced stronger IgG and IgA responses than the EV‐A71+saline group. Intranasal delivery also caused T‐cell proliferation and increased interleukin‐17 (IL‐17) levels. Antibodies from the CpG+EV‐A71 vaccine neutralized the C2 genotype and cross‐neutralized B4 and B5 genotypes [54]. A recent study explored the immune‐regulatory effects of formalin‐inactivated EVs EV‐A71 and EV‐D68, alongside PS‐G from *Ganoderma lucidum*. Mice were nasally immunized with either virus or a placebo, using PS‐G or CpG as adjuvants. The bivalent vaccine with PS‐G notably increased EV‐specific IgG and IgA levels and showed higher neutralizing antibody titers than the CpG vaccine. Intranasal vaccination produced SIgA against both viruses in mucosal areas, indicating strong immune responses without cross‐reactivity to unrelated antigens [55]. A recent study utilized immunoinformatics to identify B‐cell and T‐cell epitopes from dominant EVs such as EV‐A71, CVA16, CVA6, and CVB3, leading to the development of a novel vaccine, rCV‐A3V. An animal study confirmed the safety of intranasal vaccination, with no significant weight changes observed. The vaccine elicited a strong Th1‐biased immune response, demonstrated by high levels of serum‐neutralizing IgG and enhanced mucosal immunity, indicated by increased SIgA in nasal lavage, feces, and milk. These findings suggest that rCV‐A3V effectively promotes humoral, mucosal, and cellular immune responses against various EVs [109].

### 6.6. Challenges and Advancements in Mucosal Vaccine Development

#### 6.6.1. Route of Administration of Mucosal Vaccine

For EV mucosal vaccines, the choice of administration route is not merely a standard formulation decision; it is pivotal in determining whether protective immunity is established at the sites of viral entry and shedding. This is particularly relevant for the GIT in the cases of EV‐A71 and PV, or the respiratory tract for EV‐D68 and HRV [12, 75, 87]. Mucosal vaccine administration arises from the observation that traditional intramuscular immunization inadequately induces immunity at mucosal sites, making vaccines less effective against pathogens that use mucosal transmission [110]. During global health crises, such as pandemics, noninvasive vaccine administration methods are preferred, particularly oral and nasal routes. Oral vaccines offer benefits over injectables, such as easier self‐administration and lower cold‐chain costs for thermostable formulations. However, these advantages depend on the specific antigen, formulation, and delivery platform, rather than being inherent to the oral route itself [111]. They facilitate compliance and support rapid global vaccine deployment. A key benefit of oral vaccines is their ability to induce systemic immunity while producing antigen‐specific SIgA in the gut mucosa, providing a defense against pathogens. Additionally, they mobilize IgA‐containing lymphocytes, enhancing protection. Overall, oral vaccines offer significant advantages in controlling diseases that spread through mucosal pathways.

Oral vaccines are easy to use and effective for global deployment. They generate systemic immunity and promote SIgA in the gut, blocking pathogens. Oral vaccines mobilize IgA lymphocytes, enhancing protection, making them valuable against mucosal transmission diseases [112, 113]. Intranasal vaccine delivery avoids degradation by gastric acid and enzymes that can affect orally administered antigens. While some formulations show good systemic absorption, this varies by antigen and delivery system. This method activates localized immune responses at mucosal surfaces and allows for rapid absorption into the bloodstream. The nasal route is ideal for vaccination due to its high concentration of immune cells, which can trigger both systemic and mucosal adaptive immune responses. Additionally, the needle‐free delivery method can increase patient willingness to get vaccinated [114].

#### 6.6.2. Adjuvants for Mucosal Vaccine

The choice of adjuvant is especially important for EV mucosal vaccines because the antigens commonly employed, such as whole inactivated virus, VP1 subunit, or synthetic peptide tend to have low immunogenicity at mucosal surfaces when utilized alone. Therefore, an effective adjuvant is necessary to stimulate both IgA and cytotoxic T‐cell responses against these rapidly replicating RNA viruses [56]. Mucosal adjuvants primarily serve dual functions: they are utilized as delivery mechanisms and immunomodulatory substances. Adjuvants like chitosan and its derivatives amalgamate features of immunoenhancers and delivery platforms [115]. Research indicates that mucosal adjuvants are instrumental in invoking both localized and systemic immune defenses, pivotal for the efficacy of mucosal vaccinations against various infectious agents [116]. Beyond their immediate application, mucosal adjuvants are crucial in defending against pathogens at both proximal and distal sites of infection. For instance, cytosine‐phosphorothioate‐guanine (CpG) oligodeoxynucleotides have been identified as potent mucosal adjuvants for immunization through the nasal route, targeting diseases spread through blood transfusions and sexual intercourse [117, 118].

Additionally, their capability to enhance immune responses against neoplastic conditions delineates mucosal adjuvants as significant players in cancer immunotherapy. Among the diverse classes of mucosal adjuvants, those activating TLR and mutant enterotoxin pathways have garnered attention for their efficacy. Importantly, native CT and native *E. coli* heat‐labile enterotoxin (LT) are potent experimental adjuvants but are not considered safe for routine human mucosal vaccine use due to their toxicity; it is their detoxified mutant derivatives that show a more acceptable safety profile for potential clinical application. Predominantly, the ADP‐ribosylating enterotoxins derived from bacteria, including *Escherichia coli* heat‐LT, CT, and their LT and CT derivatives/mutants, comprise a well‐studied category of mucosal adjuvants [119]. These toxins effectively promote antigen‐specific immune responses across cellular and humoral dimensions, specifically initiating Th1, CTL, Th2, and Th17 responses. Crucially, administering mucosal routes with these adjuvants elevates antigen‐specific IgA antibody production and fosters enduring memory cell responses to vaccine antigens [120].

### 6.7. Immunization Strategy

The importance of delivery and formulation strategies for EVs is noteworthy, as many vaccine candidates, including EV‐A71 nanoparticles and lipopeptide adjuvants, remain in the preclinical phase. Thus, identifying a successful platform for stable and deployable mucosal vaccines for human use is a key challenge in advancing these vaccines [108].

Mucosal immunization is a crucial strategy for inducing mucosal protection and establishing a long‐lasting immune response as opposed to the parenteral administration of vaccines [121, 122]. The administration of vaccines via nanosized particles encapsulated within pH‐dependent microparticles for oral delivery has been explored. This innovative approach targets the large intestinal mucosal regions, leveraging the unique attributes of nanoparticles to enhance vaccine efficacy. The nanoparticle‐based oral vaccine is designed to induce antiviral immunity within both vaginal and rectal compartments, achieving a uniform level of protection akin to that of traditional colorectal vaccination methods. This strategy shows promise in offering robust defense against viral challenges in both vaginal and rectal areas, highlighting its potential as a versatile and effective vaccination technique [56]. The intranasally delivered nanoparticle‐based influenza vaccine provided protection against both similar and different strains of the virus [123]. Adjuvants for mucosal vaccines intended for human use must be combined with antigens and delivery systems. Nanoparticles are now common as mucosal carriers, showing good compatibility. Many nanoparticle vaccines are still in preclinical stages, highlighting the need for clinical trials to verify their efficacy [124]. Diet and alkalizer use are crucial for a strong mucosal immune response but may affect patient compliance. Advances in vaccine delivery systems, like mucoadhesive nanoparticles, provide promising methods for nasal and oral antigen delivery with high reproducibility [125].

## 7. Conclusion

The mucosal defense against EVs involves a complex system that includes pattern‐recognition signaling (TLR3, RIG‐I, MDA5), type I/III IFN production, and adaptive responses coordinated across various sites like GALT and NALT [1, 34]. However, the evidence base is uneven. The TLR3/IRF1/type III IFN pathway is well‐supported for EV‐A71 and PV [44, 48], but proposed pathways for other EVs such as EV‐D68 and HRV largely rely on cell line data. Additionally, much of our understanding of human mucosal immunology, such as lymphocyte density and DC movement, is extrapolated from animal models and should be seen as plausible mechanisms rather than established human physiology.

The immune evasion tactics employed by viruses further complicate the development of a cohesive model for mucosal protection. For example, EV‐A71 decreases the expression of NKG2D ligands while increasing PD‐L1 on infected IECs, thereby impeding the clearance abilities of natural killer (NK) cells and IELs [45]. Similarly, CVB3 undermines IFN signaling during its primary replication phase by depleting vital proteins such as TRIF, MAVS, and eIF4G [72]. Additionally, EV‐D68 employs at least three distinct viral proteins to cleave or inhibit components involved in the RIG‐I/MAVS and NF‐κB signaling pathways [68]. The convergence of various EVs on the same TLR3/IFN pathway through diverse molecular mechanisms suggests that this pathway is a promising target for vaccines and antiviral therapies. However, it also indicates that a uniform mucosal vaccine platform may not be uniformly effective against the genetically and antigenically varied EVs. Among existing licensed mucosal vaccines, the OPV and the rotavirus vaccines provide evidence that oral immunization can confer population‐level protection against enteric viral diseases, as evidenced by a more than 99% reduction in global PV incidence since the 1980 s [92] and the significant decrease in rotavirus‐related mortality and hospitalizations enabled by Rotarix and RotaTeq across numerous countries [92]. Nonetheless, these examples illustrate that live mucosal vaccines are associated with real, nontheoretical risks, such as the emergence of vaccine‐derived PV and a low, yet significant, rate of intussusception, which necessitates thorough post‐marketing surveillance rather than being regarded as resolved issues [100].

In contrast, mucosal vaccine candidates for EV‐A71 and other NPEVs are still at the preclinical stage. Various platforms, including nanoparticles, lipopeptides, and CpG–based adjuvants, have shown promising IgA, IgG, and T‐cell responses [54, 107, 109]. However, these studies were conducted using inbred rodent models and homologous challenge strains, and none has progressed to human clinical trials. Therefore, drawing conclusions about the potential for human protection from these models is premature. This is particularly true since the mucosal adjuvants that have demonstrated the most experimental activity, such as native CT and *E. coli* heat‐LT, are not suitable for direct human use; their detoxified versions still need to show comparable immunogenicity in humans [121]. The development of safe and effective adjuvants for human mucosal vaccination remains one of the most significant unresolved challenges in the field [104–106]. To advance this area, it will be essential to conduct mechanistic studies using native human mucosal tissues, rather than solely relying on immortalized cell lines or animal models [125]. It is also critical to develop delivery platforms that are both immunogenic and suitable for routine use in humans, alongside initiating early‐phase clinical trials to assess if preclinical mucosal immunogenicity results in actual human protection. Until these advancements are made, the clinical benefits of mucosal vaccination against NPEVs will remain a plausible hypothesis rather than a confirmed conclusion.

## 8. Limitations

This review has several limitations to consider when interpreting its conclusions. First, it is a narrative review rather than a systematic one, lacking a predefined search protocol, dual‐reviewer screening, or formal risk‐of‐bias assessment, which could introduce selection bias. Second, the underlying evidence base is highly heterogeneous: mechanistic claims for EV‐A71 and CVA16 largely depend on immortalized cell line models like HT‐29 and isolated mechanistic studies of tuft cell biology, limiting generalizability to native human mucosal tissue. Third, the contributions of individual innate signaling pathways proposed for EV‐D68 and HRV have largely been inferred from in vitro airway epithelial models instead of validated in vivo, leaving their true hierarchy unclear. Fourth, echovirus 11 findings come from a single sibling case comparison, and the inference that mucosal IFN deficiency drives systemic dissemination is more of a hypothesis than an established fact. Fifth, the review is biased toward innate and humoral mucosal responses, with only sparse discussion of adaptive Trm immunity in GALT and NALT, highlighting a gap in the literature. Finally, most mucosal vaccine candidates have only been evaluated in animal models, making their advantages over parenteral vaccination in humans provisional pending clinical validation.

## Author Contributions

Nida Kalam conceptualized, designed, and wrote the manuscript. Nida Kalam designed all the figures and critically revised the manuscript. Vinod R. M. T. Balasubramaniam provided critical review and supervision.

## Funding

We did not receive any specific funding for this work. Open access publishing facilitated by Monash University, as part of the Wiley ‐ Monash University agreement via the Council of Australasian University Librarians.

## Disclosure

All the authors approved the final version of the manuscript.

## Conflicts of Interest

The authors declare no conflicts of interest.

## Data Availability

This is a literature review paper. There is no new data generated in this article.

## References

[1] Zhou X. , Wu Y. , and Zhu Z. , et al.Mucosal Immune Response in Biology, Disease Prevention and Treatment, Signal Transduction and Targeted Therapy. (2025) 10, no. 1, 10.1038/s41392-024-02043-4, 7. 39774607 PMC11707400

[2] Zhang Z. , Hong W. , Zhang Y. , Li X. , Que H. , and Wei X. , Mucosal Immunity and Vaccination Strategies: Current Insights and Future Perspectives, Molecular Biomedicine. (2025) 6, no. 1, 10.1186/s43556-025-00301-7, 57. 40830509 PMC12364801

[3] Moradi-kalbolandi S. , Majidzadeh-A K. , Abdolvahab M. H. , Jalili N. , and Farahmand L. , The Role of Mucosal Immunity and Recombinant Probiotics in SARS-CoV2 Vaccine Development, Probiotics and Antimicrobial Proteins. (2021) 13, no. 5, 1239–1253, 10.1007/s12602-021-09773-9. 33770348 PMC7996120

[4] Yi E.-J. , Kim Y.-I. , Song J.-H. , Ko H.-J. , and Chang S.-Y. , Intranasal Immunization With Curdlan Induce Th17 Responses and Enhance Protection Against Enterovirus 71, Vaccine. (2023) 41, no. 13, 2243–2252, 10.1016/j.vaccine.2023.01.074. 36863926

[5] Godin A. , Brickley E. B. , and Connor R. I. , et al.Intestinal Mucosal Immune Responses to Novel Oral Poliovirus Vaccine Type 2 in Healthy Newborns, Clinical Infectious Diseases. (2026) 82, no. 2, e352–e360, 10.1093/cid/ciaf484. 40907970 PMC13017628

[6] Wells A. I. and Coyne C. B. , Enteroviruses: A Gut-Wrenching Game of Entry, Detection, and Evasion, Viruses. (2019) 11, no. 5, 10.3390/v11050460, 460. 31117206 PMC6563291

[7] Paludan S. R. and Bowie A. G. , Immune Sensing of DNA, Immunity. (2013) 38, no. 5, 870–880, 10.1016/j.immuni.2013.05.004. 23706668 PMC3683625

[8] Aoshi T. , Koyama S. , Kobiyama K. , Akira S. , and Ishii K. J. , Innate and Adaptive Immune Responses to Viral Infection and Vaccination, Current Opinion in Virology. (2011) 1, no. 4, 226–232, 10.1016/j.coviro.2011.07.002. 22440781

[9] Triantafilou K. , Vakakis E. , Richer E. A. J. , Evans G. L. , Villiers J. P. , and Triantafilou M. , Human Rhinovirus Recognition in Non-Immune Cells Is Mediated by *Toll*-Like Receptors and MDA-5, Which Trigger a Synergetic pro-Inflammatory Immune Response, Virulence. (2011) 2, no. 1, 22–29, 10.4161/viru.2.1.13807. 21224721 PMC3073236

[10] Chen K. R. , Yu C. K. , and Kung S. H. , et al.Toll-Like Receptor 3 Is Involved in Detection of Enterovirus A71 Infection and Targeted by Viral 2A Protease, Viruses. (2018) 10, no. 12, 10.3390/v10120689, 689. 30563052 PMC6315976

[11] García-Sastre A. , Ten Strategies of Interferon Evasion by Viruses, Cell Host & Microbe. (2017) 22, no. 2, 176–184, 10.1016/j.chom.2017.07.012. 28799903 PMC5576560

[12] Chen K.-R. and Ling P. , Interplays Between Enterovirus A71 and the Innate Immune System, Journal of Biomedical Science. (2019) 26, no. 1, 10.1186/s12929-019-0596-8, 95. 31787104 PMC6886175

[13] Linden S. K. , Sutton P. , Karlsson N. G. , Korolik V. , and McGuckin M. A. , Mucins in the Mucosal Barrier to Infection, Mucosal Immunology. (2008) 1, no. 3, 183–197, 10.1038/mi.2008.5. 19079178 PMC7100821

[14] Brandtzaeg P. , Kiyono H. , Pabst R. , and Russell M. W. , Terminology: Nomenclature of Mucosa-Associated Lymphoid Tissue, Mucosal Immunology. (2008) 1, no. 1, 31–37, 10.1038/mi.2007.9. 19079158

[15] van Wijk F. and Cheroutre H. , Intestinal T Cells: Facing the Mucosal Immune Dilemma With Synergy and Diversity, Seminars in Immunology. (2009) 21, no. 3, 130–138, 10.1016/j.smim.2009.03.003. 19386513 PMC2794834

[16] Olivares-Villagómez D. and Van Kaer L. , Intestinal Intraepithelial Lymphocytes: Sentinels of the Mucosal Barrier, Trends in Immunology. (2018) 39, no. 4, 264–275, 10.1016/j.it.2017.11.003. 29221933 PMC8056148

[17] Brandtzaeg P. and Johansen F. E. , Mucosal B Cells: Phenotypic Characteristics, Transcriptional Regulation, and Homing Properties, Immunological Reviews. (2005) 206, no. 1, 32–63, 10.1111/j.0105-2896.2005.00283.x. 16048541

[18] Fagarasan S. and Honjo T. , Intestinal IgA Synthesis: Regulation of Front-Line Body Defences, Nature Reviews Immunology. (2003) 3, no. 1, 63–72, 10.1038/nri982. 12511876

[19] Brandtzaeg P. and Pabst R. , Let’s Go Mucosal: Communication on Slippery Ground, Trends in Immunology. (2004) 25, no. 11, 570–577, 10.1016/j.it.2004.09.005. 15489184

[20] Coombes J. L. , Siddiqui K. R. , and Arancibia-Cárcamo C. V. , et al.A Functionally Specialized Population of Mucosal CD103^+^ DCs Induces Foxp3^+^ Regulatory T Cells via a TGF-β– and Retinoic Acid–Dependent Mechanism, The Journal of Experimental Medicine. (2007) 204, no. 8, 1757–1764, 10.1084/jem.20070590. 17620361 PMC2118683

[21] Laffont S. , Siddiqui K. R. R. , and Powrie F. , Intestinal Inflammation Abrogates the Tolerogenic Properties of MLN CD103^+^ Dendritic Cells, European Journal of Immunology. (2010) 40, no. 7, 1877–1883, 10.1002/eji.200939957. 20432234 PMC6108414

[22] Powrie F. , Leach M. W. , Mauze S. , Menon S. , Caddle L. B. , and Coffman R. L. , Inhibition of Th1 Responses Prevents Inflammatory Bowel Disease in Scid Mice Reconstituted With CD45RBhi CD4^+^ T Cells, Immunity. (1994) 1, no. 7, 553–562, 10.1016/1074-7613(94)90045-0. 7600284

[23] Simpson S. and Wetzler L. M. , Pier G. B. , Lyczak J. B. , and Wetzler L. M. , Mucosal Immunity. In Immunology, Infection, and Immunity, 2004, John Wiley & Sons, 399–423, 10.1128/9781555816148.ch17.

[24] Maldonado-Contreras A. L. and McCormick B. A. , Intestinal Epithelial Cells and Their Role in Innate Mucosal Immunity, Cell and Tissue Research. (2011) 343, no. 1, 5–12, 10.1007/s00441-010-1082-5. 21104188 PMC4131254

[25] Wu R.-Q. , Zhang D.-F. , Tu E. , Chen Q.-M. , and Chen W. J. , The Mucosal Immune System in the Oral Cavity—an Orchestra of T Cell Diversity, International Journal of Oral Science. (2014) 6, no. 3, 125–132, 10.1038/ijos.2014.48. 25105816 PMC4170154

[26] Mörbe U. M. , Jørgensen P. B. , and Fenton T. M. , et al.Human Gut-Associated Lymphoid Tissues (GALT); Diversity, Structure, and Function, Mucosal Immunology. (2021) 14, no. 4, 793–802, 10.1038/s41385-021-00389-4. 33753873

[27] Fries P. N. , Popowych Y. I. , Guan L. L. , and Griebel P. J. , Age-Related Changes in the Distribution and Frequency of Myeloid and T Cell Populations in the Small Intestine of Calves, Cellular Immunology. (2011) 271, no. 2, 428–437, 10.1016/j.cellimm.2011.08.012. 21917242

[28] Yi A.-K. , Yoon J.-G. , Yeo S.-J. , Hong S.-C. , English B. K. , and Krieg A. M. , Role of Mitogen-Activated Protein Kinases in CpG DNA-Mediated IL-10 and IL-12 Production: Central Role of Extracellular Signal-Regulated Kinase in the Negative Feedback Loop of the CpG DNA-Mediated Th1 Response, The Journal of Immunology. (2002) 168, no. 9, 4711–4720, 10.4049/jimmunol.168.9.4711. 11971021

[29] Kataoka K. , McGhee J. R. , Kobayashi R. , Fujihashi K. , Shizukuishi S. , and Fujihashi K. , Nasal Flt3 Ligand cDNA Elicits CD11c^+^CD8^+^ Dendritic Cells for Enhanced Mucosal Immunity, The Journal of Immunology. (2004) 172, no. 6, 3612–3619, 10.4049/jimmunol.172.6.3612. 15004163

[30] Chang S. Y. , Cha H. R. , and Igarashi O. , et al.Cutting Edge: Langerin+ Dendritic Cells in the Mesenteric Lymph Node Set the Stage for Skin and Gut Immune System Cross-Talk, The Journal of Immunology. (2008) 180, no. 7, 4361–4365, 10.4049/jimmunol.180.7.4361. 18354155

[31] Flores-Langarica A. , Marshall J. L. , and Hitchcock J. , et al.Systemic Flagellin Immunization Stimulates Mucosal CD103^+^ Dendritic Cells and Drives Foxp3^+^ Regulatory T Cell and IgA Responses in the Mesenteric Lymph Node, The Journal of Immunology. (2012) 189, no. 12, 5745–5754, 10.4049/jimmunol.1202283. 23152564

[32] Ozawa Y. , Suda T. , and Nagata T. , et al.Mucosal Vaccine Using CTL Epitope-Pulsed Dendritic Cell Confers Protection for Intracellular Pathogen, American Journal of Respiratory Cell and Molecular Biology. (2009) 41, no. 4, 440–448, 10.1165/rcmb.2008-0446OC. 19202004

[33] Pietrzak B. , Tomela K. , Olejnik-Schmidt A. , Mackiewicz A. , and Schmidt M. , Secretory IgA in Intestinal Mucosal Secretions as an Adaptive Barrier Against Microbial Cells, International Journal of Molecular Sciences. (2020) 21, no. 23, 10.3390/ijms21239254, 9254. 33291586 PMC7731431

[34] Mantis N. J. , Rol N. , and Corthésy B. , Secretory IgA’s Complex Roles in Immunity and Mucosal Homeostasis in the Gut, Mucosal Immunology. (2011) 4, no. 6, 603–611, 10.1038/mi.2011.41. 21975936 PMC3774538

[35] Zhaori G. , Sun M. , Faden H. S. , and Ogra P. L. , Nasopharyngeal Secretory Antibody Response to Poliovirus Type 3 Virion Proteins Exhibit Different Specificities After Immunization With Live or Inactivated Poliovirus Vaccines, Journal of Infectious Diseases. (1989) 159, no. 6, 1018–1024, 10.1093/infdis/159.6.1018. 2470831

[36] Park J. G. , Alfajaro M. M. , and Cho E. H. , et al.Development of a Live Attenuated Trivalent Porcine Rotavirus a Vaccine Against Disease Caused by Recent Strains Most Prevalent in South Korea, Veterinary Research. (2019) 50, no. 1, 10.1186/s13567-018-0619-6, 2. 30616694 PMC6323864

[37] Takeuchi O. and Akira S. , Pattern Recognition Receptors and Inflammation, Cell. (2010) 140, no. 6, 805–820, 10.1016/j.cell.2010.01.022. 20303872

[38] Holm C. K. , Paludan S. R. , and Fitzgerald K. A. , DNA Recognition in Immunity and Disease, Current Opinion in Immunology. (2013) 25, no. 1, 13–18, 10.1016/j.coi.2012.12.006. 23313533 PMC3645908

[39] Mogensen T. H. , Pathogen Recognition and Inflammatory Signaling in Innate Immune Defenses, Clinical Microbiology Reviews. (2009) 22, no. 2, 240–273, 10.1128/CMR.00046-08. 19366914 PMC2668232

[40] Chow J. , Franz K. M. , and Kagan J. C. , PRRs Are Watching You: Localization of Innate Sensing and Signaling Regulators, Virology. (2015) 479–480, 104–109, 10.1016/j.virol.2015.02.051. PMC4424080 25800355

[41] Kawai T. and Akira S. , Toll-Like Receptors and Their Crosstalk With Other Innate Receptors in Infection and Immunity, Immunity. (2011) 34, no. 5, 637–650, 10.1016/j.immuni.2011.05.006. 21616434

[42] Lei X. , Liu X. , and Ma Y. , et al.The 3C Protein of Enterovirus 71 Inhibits Retinoid Acid-Inducible Gene I-Mediated Interferon Regulatory Factor 3 Activation and Type I Interferon Responses, Journal of Virology. (2010) 84, no. 16, 8051–8061, 10.1128/JVI.02491-09. 20519382 PMC2916543

[43] Wedekind A. , Fritzsch D. , and Kröger A. , Add on the Next Level—the Time Point of the Type I IFN Response Orchestrates the Immune Response, Cellular & Molecular Immunology. (2020) 17, no. 8, 791–793, 10.1038/s41423-020-0442-7. 32346100 PMC7186764

[44] Su R. , Shereen M. A. , and Zeng X. , et al.The TLR3/IRF1/Type III IFN Axis Facilitates Antiviral Responses Against Enterovirus Infections in the Intestine, mBio. (2020) 11, no. 6, 10.1128/mBio.02540-20. PMC7683398 33203755

[45] Dong Y. , Liu J. , Lu N. , and Zhang C. , Enterovirus 71 Antagonizes Antiviral Effects of Type III Interferon and Evades the Clearance of Intestinal Intraepithelial Lymphocytes, Frontiers in Microbiology. (2022) 12, 10.3389/fmicb.2021.806084, 806084. 35185830 PMC8848745

[46] Sethi Y. , Kaka N. , Chopra H. , Aggarwal N. , Arora S. , and Emran T. B. , Recent Poliovirus Outbreaks and Vaccination: A Perspective, Annals of Medicine & Surgery. (2022) 84, 10.1016/j.amsu.2022.104970, 104970. 36439891 PMC9682333

[47] Iwasaki A. , Welker R. , Mueller S. , Linehan M. , Nomoto A. , and Wimmer E. , Immunofluorescence Analysis of Poliovirus Receptor Expression in Peyer’s Patches of Humans, Primates, and CD155 Transgenic Mice: Implications for Poliovirus Infection, The Journal of Infectious Diseases. (2002) 186, no. 5, 585–592, 10.1086/342682. 12195344

[48] Oshiumi H. , Okamoto M. , and Fujii K. , et al.The TLR3/TICAM-1 Pathway Is Mandatory for Innate Immune Responses to Poliovirus Infection, The Journal of Immunology. (2011) 187, no. 10, 5320–5327, 10.4049/jimmunol.1101503. 21998457

[49] McMinn P. C. , An Overview of the Evolution of Enterovirus 71 and Its Clinical and Public Health Significance, FEMS Microbiology Reviews. (2002) 26, no. 1, 91–107, 10.1111/j.1574-6976.2002.tb00601.x. 12007645

[50] Hwang K. P. , Luong Q. C. , and Huang Y. C. , et al.A Randomised Trial of a Bioreactor-Produced EV-A71 Vaccine for Endemic Control in Children, npj Vaccines. (2026) 11, 10.1038/s41541-026-01396-x, 65. 41680199 PMC13009275

[51] Ong K. C. and Wong K. T. , Understanding Enterovirus 71 Neuropathogenesis and Its Impact on Other Neurotropic Enteroviruses, Brain Pathology. (2015) 25, no. 5, 614–624, 10.1111/bpa.12279. 26276025 PMC8029433

[52] Cox J. A. , Hiscox J. A. , Solomon T. , Ooi M.-H. , and Ng L. F. P. , Immunopathogenesis and Virus–Host Interactions of Enterovirus 71 in Patients With Hand, Foot and Mouth Disease, Frontiers in Microbiology. (2017) 8, 10.3389/fmicb.2017.02249, 2249. 29238324 PMC5713468

[53] Lui Y. L. E. , Timms P. , Hafner L. M. , Tan T. L. , Tan K. H. , and Tan E. L. , Characterisation of Enterovirus 71 Replication Kinetics in Human Colorectal Cell Line, HT29, SpringerPlus. (2013) 2, no. 1, 10.1186/2193-1801-2-267, 267. 23875129 PMC3696168

[54] Lin Y. L. , Chow Y. H. , and Huang L. M. , et al.A CpG-Adjuvanted Intranasal Enterovirus 71 Vaccine Elicits Mucosal and Systemic Immune Responses and Protects Human SCARB2-Transgenic Mice Against Lethal Challenge, Scientific Reports. (2018) 8, no. 1, 10.1038/s41598-018-28281-5, 10713. 30013088 PMC6048030

[55] Lin Y.-L. , Cheng P.-Y. , and Chin C.-L. , et al.A Novel Mucosal Bivalent Vaccine of EV-A71/EV-D68 Adjuvanted With Polysaccharides From *Ganoderma lucidum* Protects Mice Against EV-A71 and EV-D68 Lethal Challenge, Journal of Biomedical Science. (2023) 30, no. 1, 10.1186/s12929-023-00987-3, 96. 38110940 PMC10729491

[56] Zhang F. , Hao C. , and Zhang S. , et al.Oral Immunization With Recombinant Enterovirus 71 VP1 Formulated With Chitosan Protects Mice Against Lethal Challenge, Virology Journal. (2014) 11, no. 1, 10.1186/1743-422X-11-80, 80. 24885121 PMC4022980

[57] Zhang X. , Shi J. , and Ye X. , et al.Coxsackievirus A16 Utilizes Cell Surface Heparan Sulfate Glycosaminoglycans as Its Attachment Receptor, Emerging Microbes & Infections. (2017) 6, no. 1, 10.1038/emi.2017.55. PMC5567171 28745308

[58] Fu X. , Wan Z. , Li Y. , Hu Y. , Jin X. , and Zhang C. , National Epidemiology and Evolutionary History of Four Hand, Foot and Mouth Disease-Related Enteroviruses in China From 2008 to 2016, Virologica Sinica. (2020) 35, no. 1, 21–33, 10.1007/s12250-019-00169-2. 31664644 PMC7035399

[59] Mao Q. , Wang Y. , and Yao X. , et al.Coxsackievirus A16: Epidemiology, Diagnosis, and Vaccine, Human Vaccines & Immunotherapeutics. (2014) 10, no. 2, 360–367, 10.4161/hv.27087. 24231751 PMC4185891

[60] Liu Y. , Maisimu M. , Ge Z. , Xiao S. , and Wang H. , The Pathogenesis and Virulence of the Major Enterovirus Pathogens Associated With Severe Clinical Manifestations: A Comprehensive Review, Cells. (2025) 14, no. 20, 10.3390/cells14201617, 1617. 41148832 PMC12562741

[61] Rui Y. , Su J. , and Wang H. , et al.Disruption of MDA5-Mediated Innate Immune Responses by the 3C Proteins of Coxsackievirus A16, Coxsackievirus A6, and Enterovirus D68, Journal of Virology. (2017) 91, no. 13, 10.1128/JVI.00546-17. PMC5469270 28424289

[62] Chen D. , Wu J. , and Zhang F. , et al.Trained Immunity of Intestinal Tuft Cells During Infancy Enhances Host Defense Against Enteroviral Infections in Mice, EMBO Molecular Medicine. (2024) 16, no. 10, 2516–2538, 10.1038/s44321-024-00128-9. 39261649 PMC11479266

[63] Chen X. , Zhang Y. , Mao N. , Zhu S. , Ji T. , and Xu W. , Intranasal Immunization With Coxsackievirus A16 Virus-Like Particles Confers Protection Against Lethal Infection in Neonatal Mice, Archives of Virology. (2019) 164, no. 12, 2975–2984, 10.1007/s00705-019-04418-3. 31570994

[64] Bragstad K. , Jakobsen K. , and Rojahn A. E. , et al.High Frequency of Enterovirus D68 in Children Hospitalised With Respiratory Illness in Norway, Autumn 2014, Influenza Other Respir Viruses. (2015) 9, no. 2, 59–63. 25534826 10.1111/irv.12300 PMC4353317

[65] Esposito S. , Zampiero A. , Ruggiero L. , Madini B. , Niesters H. , and Principi N. , Enterovirus D68-Associated Community-Acquired Pneumonia in Children Living in Milan, Italy, Journal of Clinical Virology. (2015) 68, 94–96, 10.1016/j.jcv.2015.05.017. 26071345

[66] Freeman M. C. , Wells A. I. , and Ciomperlik-Patton J. , et al.Respiratory and Intestinal Epithelial Cells Exhibit Differential Susceptibility and Innate Immune Responses to Contemporary EV-D68 Isolates, eLife. (2021) 10, 10.7554/eLife.66687. PMC8285104 34196272

[67] Naeem A. , Bello M. B. , and Bosaeed M. , Insights Into Enterovirus D68 Immunology: Unraveling the Mysteries of Host-Pathogen Interactions, Immunity, Inflammation and Disease. (2025) 13, no. 2, 10.1002/iid3.70117. PMC11800235 39912556

[68] Kang J. , Huang M. , and Li J. , et al.Enterovirus D68 VP3 Targets the Interferon Regulatory Factor 7 To Inhibit Type I Interferon Response, Microbiology Spectrum. (2023) 11, no. 3, e04138–e04122, 10.1128/spectrum.04138-22. 37125923 PMC10269600

[69] Chio C.-C. , Chan H.-W. , Chen S.-H. , and Huang H.-I. , Enterovirus D68 vRNA Induces Type III IFN Production via MDA5, Virus Research. (2024) 339, 10.1016/j.virusres.2023.199284, 199284. 38040125 PMC10704515

[70] Bergelson J. M. , Cunningham J. A. , and Droguett G. , et al.Isolation of a Common Receptor for Coxsackie B Viruses and Adenoviruses 2 and 5, Science. (1997) 275, no. 5304, 1320–1323, 10.1126/science.275.5304.1320. 9036860

[71] Shieh J. T. C. and Bergelson J. M. , Interaction With Decay-Accelerating Factor Facilitates Coxsackievirus B Infection of Polarized Epithelial Cells, Journal of Virology. (2002) 76, no. 18, 9474–9480, 10.1128/JVI.76.18.9474-9480.2002. 12186929 PMC136423

[72] Stone V. M. , Ringqvist E. E. , and Larsson P. G. , et al.Inhibition of Type III Interferon Expression in Intestinal Epithelial Cells—A Strategy Used by Coxsackie B Virus to Evade the Host’s Innate Immune Response at the Primary Site of Infection?, Microorganisms. (2021) 9, no. 1, 10.3390/microorganisms9010105, 105. 33466313 PMC7824802

[73] Hyöty H. , Kääriäinen S. , and Laiho J. E. , et al.Safety, Tolerability and Immunogenicity of PRV-101, a Multivalent Vaccine Targeting Coxsackie B Viruses (CVBs) Associated With Type 1 Diabetes: A Double-Blind Randomised Placebo-Controlled Phase I Trial, Diabetologia. (2024) 67, no. 5, 811–821, 10.1007/s00125-024-06092-w. 38369573 PMC10954874

[74] Atmar R. L. , Editorial Commentary: Uncommon(ly Considered) Manifestations of Infection With Rhinovirus, Agent of the Common Cold, Clinical Infectious Diseases. (2005) 41, no. 2, 266–267, 10.1086/430927. 15983927

[75] Proud D. , Hudy M. H. , and Wiehler S. , et al.Cigarette Smoke Modulates Expression of Human Rhinovirus-Induced Airway Epithelial Host Defense Genes, PLoS ONE. (2012) 7, no. 7, 10.1371/journal.pone.0040762. PMC3395625 22808255

[76] Khaitov M. R. , Laza-Stanca V. , and Edwards M. R. , et al.Respiratory Virus Induction of Alpha-, Beta- and Lambda-Interferons in Bronchial Epithelial Cells and Peripheral Blood Mononuclear Cells, Allergy. (2009) 64, no. 3, 375–386, 10.1111/j.1398-9995.2008.01826.x. 19175599

[77] Warner S. M. , Wiehler S. , Michi A. N. , and Proud D. , Rhinovirus Replication and Innate Immunity in Highly Differentiated Human Airway Epithelial Cells, Respiratory Research. (2019) 20, no. 1, 10.1186/s12931-019-1120-0, 150. 31299975 PMC6626354

[78] Wimmers F. , Burrell A. R. , and Feng Y. , et al.Multi-Omics Analysis of Mucosal and Systemic Immunity to SARS-CoV-2 After Birth, Cell. (2023) 186, no. 21, 4632–4651.e23, 10.1016/j.cell.2023.08.044. 37776858 PMC10724861

[79] Wang B. , Amat J. A. R. , Mihaylova V. T. , Kong Y. , Wang G. , and Foxman E. F. , Rhinovirus Triggers Distinct Host Responses Through Differential Engagement of Epithelial Innate Immune Signaling, Cell Press Blue. (2026) 1, no. 1, 10.1016/j.cpblue.2025.100001, 100001.

[80] Steinke J. W. , Liu L. , Turner R. B. , Braciale T. J. , and Borish L. , Immune Surveillance by Rhinovirus-Specific Circulating CD4^+^ and CD8^+^ T Lymphocytes, PLoS ONE. (2015) 10, no. 1, 10.1371/journal.pone.0115271. PMC4293146 25584821

[81] Price A. S. and Kennedy J. L. , T-Helper 2 Mechanisms Involved in Human Rhinovirus Infections and Asthma, Annals of Allergy, Asthma & Immunology. (2022) 129, no. 6, 681–691, 10.1016/j.anai.2022.08.015. PMC10316285 36002092

[82] Brandtzaeg P. , Induction of Secretory Immunity and Memory at Mucosal Surfaces, Vaccine. (2007) 25, no. 30, 5467–5484, 10.1016/j.vaccine.2006.12.001. 17227687

[83] Igarashi Y. , Skoner D. P. , Doyle W. J. , White M. V. , Fireman P. , and Kaliner M. A. , Analysis of Nasal Secretions During Experimental Rhinovirus Upper Respiratory Infections, Journal of Allergy and Clinical Immunology. (1993) 92, no. 5, 722–731, 10.1016/0091-6749(93)90016-9. 8227864

[84] Iheukwumere I. H. , Iheukwumere C. M. , Unaeze B. C. , Ike V. E. , Nnadozie H. C. , and Onyema S. O. , Human Echovirus Infection: Virology, Pathogenesis, Clinical Manifestations, and Management, IPS Journal of Applied Microbiology and Biotechnology. (2025) 4, no. 4, 203–209, 10.54117/ijamb.v4i4.91.

[85] Funaki T. and Shoji K. , The Current Status of Neonatal Echovirus 11 Infections in Japan: A Comparison to the Global Situation, Journal of Infection and Chemotherapy. (2026) 32, no. 1, 10.1016/j.jiac.2025.102879, 102879. 41338390

[86] Novazzi F. , Piralla A. , and Perniciaro S. , et al.A New Case of Echovirus 11 Neonatal Fulminant Hepatitis Involving Male Twins in a Northern Italy Tertiary University Hospital: Insight on a Possible Immunological Clue, IJID Regions. (2024) 12, 10.1016/j.ijregi.2024.100411, 100411. 39220203 PMC11363566

[87] Wright P. F. , Wieland-Alter W. , and Ilyushina N. A. , et al.Intestinal Immunity Is a Determinant of Clearance of Poliovirus After Oral Vaccination, The Journal of Infectious Diseases. (2014) 209, no. 10, 1628–1634, 10.1093/infdis/jit671. 24459191

[88] Herremans T. M. P. T. , Reimerink J. H. J. , Buisman A. M. , Kimman T. G. , and Koopmans M. P. G. , Induction of Mucosal Immunity by Inactivated Poliovirus Vaccine Is Dependent on Previous Mucosal Contact With Live Virus, The Journal of Immunology. (1999) 162, no. 8, 5011–5018, 10.4049/jimmunol.162.8.5011. 10202050

[89] Baicus A. , History of Polio Vaccination, World Journal of Virology. (2012) 1, no. 4, 108–114, 10.5501/wjv.v1.i4.108. 24175215 PMC3782271

[90] Hampton L. M. , Farrell M. , and Ramirez-Gonzalez A. , et al.Cessation of Trivalent Oral Poliovirus Vaccine and Introduction of Inactivated Poliovirus Vaccine—Worldwide, 2016, MMWR. Morbidity and Mortality Weekly Report. (2016) 65, no. 35, 934–938, 10.15585/mmwr.mm6535a3. 27606675

[91] Ward N. and Hull H. , Polio eradication, The Lancet. (1995) 345, no. 8945, 318. 10.1016/s0140-6736(95)90305-4 7837876

[92] Jiang B. , Patel M. , and Glass R. I. , Polio Endgame: Lessons for the Global Rotavirus Vaccination Program, Vaccine. (2019) 37, no. 23, 3040–3049, 10.1016/j.vaccine.2019.04.023. 31027927

[93] De Coster I. , Leroux-Roels I. , and Bandyopadhyay A. S. , et al.Safety and Immunogenicity of Two Novel Type 2 Oral Poliovirus Vaccine Candidates Compared With a Monovalent Type 2 Oral Poliovirus Vaccine in Healthy Adults: Two Clinical Trials, The Lancet. (2021) 397, no. 10268, 39–50, 10.1016/S0140-6736(20)32541-1. PMC7811203 33308429

[94] Zhao T. , Li J. , and Shi H. , et al.Reduced Mucosal Immunity to Poliovirus After Cessation of Trivalent Oral Polio Vaccine, Human Vaccines & Immunotherapeutics. (2021) 17, no. 8, 2560–2567, 10.1080/21645515.2021.1911213. 33848232 PMC8475588

[95] Connor R. I. , Brickley E. B. , and Wieland-Alter W. F. , et al.Mucosal Immunity to Poliovirus, Mucosal Immunology. (2022) 15, no. 1, 1–9, 10.1038/s41385-021-00428-0. 34239028 PMC8732262

[96] Patel M. , Shane A. L. , Parashar U. D. , Jiang B. , Gentsch J. R. , and Glass R. I. , Oral Rotavirus Vaccines: How Well Will They Work Where They Are Needed Most?, The Journal of Infectious Diseases. (2009) 200, no. s1, S39–S48, 10.1086/605035. 19817613 PMC3673012

[97] Glass R. I. , Parashar U. D. , and Bresee J. S. , et al.Rotavirus Vaccines: Current Prospects and Future Challenges, Lancet. (2006) 368, no. 9532, 323–332, 10.1016/S0140-6736(06)68815-6. 16860702

[98] Clark A. , Black R. , and Tate J. , et al.Estimating Global, Regional and National Rotavirus Deaths in Children Aged <5 Years: Current Approaches, New Analyses and Proposed Improvements, PLoS ONE. (2017) 12, no. 9, 10.1371/journal.pone.0183392. PMC5593200 28892480

[99] Abou-Nader A. J. , Sauer M. A. , and Steele A. D. , et al.Global Rotavirus Vaccine Introductions and Coverage: 2006–2016, Human Vaccines & Immunotherapeutics. (2018) 14, no. 9, 2281–2296. 29787334 10.1080/21645515.2018.1470725 PMC6183203

[100] Yen C. , Healy K. , and Tate J. E. , et al.Rotavirus Vaccination and Intussusception—Science, Surveillance, and Safety: A Review of Evidence and Recommendations for Future Research Priorities in Low and Middle Income Countries, Human Vaccines & Immunotherapeutics. (2016) 12, no. 10, 2580–2589, 10.1080/21645515.2016.1197452. 27322835 PMC5084992

[101] Burnett E. , Parashar U. D. , and Tate J. E. , Real-World Effectiveness of Rotavirus Vaccines, 2006–19: A Literature Review and Meta-Analysis, The Lancet Global Health. (2020) 8, no. 9, e1195–e1202, 10.1016/S2214-109X(20)30262-X. 32827481 PMC8097518

[102] Baker J. M. , Dahl R. M. , Cubilo J. , Parashar U. D. , and Lopman B. A. , Effects of the Rotavirus Vaccine Program Across Age Groups in the United States: Analysis of National Claims Data, 2001–2016, BMC Infectious Diseases. (2019) 19, no. 1, 10.1186/s12879-019-3816-7, 186. 30795739 PMC6387516

[103] Middleton B. F. , Fathima P. , Snelling T. L. , and Morris P. , Systematic Review of the Effect of Additional Doses of Oral Rotavirus Vaccine on Immunogenicity and Reduction in Diarrhoeal Disease Among Young Children, eClinicalMedicine. (2022) 54, 10.1016/j.eclinm.2022.101687, 101687. 36247922 PMC9561686

[104] Song Y. , Mehl F. , and Zeichner S. L. , Vaccine Strategies to Elicit Mucosal Immunity, Vaccines. (2024) 12, no. 2, 10.3390/vaccines12020191, 191. 38400174 PMC10892965

[105] Huang H.-I. , Chio C.-C. , Lin J.-Y. , and Xing Z. , Inhibition of EV71 by Curcumin in Intestinal Epithelial Cells, PLoS ONE. (2018) 13, no. 1, 10.1371/journal.pone.0191617. PMC5784943 29370243

[106] Anggraeni R. , Ana I. D. , and Wihadmadyatami H. , Development of Mucosal Vaccine Delivery: An Overview on the Mucosal Vaccines and Their Adjuvants, Clinical and Experimental Vaccine Research. (2022) 11, no. 3, 235–248, 10.7774/cevr.2022.11.3.235. 36451668 PMC9691869

[107] Saeed M. I. , Omar A. R. , Hussein M. Z. , Elkhidir I. M. , and Sekawi Z. , Development of Enhanced Antibody Response Toward Dual Delivery of Nano-Adjuvant Adsorbed Human *Enterovirus-71* Vaccine Encapsulated Carrier, Human Vaccines & Immunotherapeutics. (2015) 11, no. 10, 2414–2424, 10.1080/21645515.2015.1052918. 26186664 PMC4635880

[108] Lin Y.-L. , Cheng P.-Y. , Chin C.-L. , Huang L.-M. , Lin S.-Y. , and Chiang B.-L. , Fibroblast-Stimulating Lipopeptide-1 as a Potential Mucosal Adjuvant Enhances Mucosal and Systemic Immune Responses to Enterovirus 71 Vaccine, Vaccine. (2018) 36, no. 29, 4331–4338, 10.1016/j.vaccine.2018.05.090. 29891349

[109] Zheng J. , Deng H. , and Liu Z. , et al.Construction of the Multi-Epitope HFMD Vaccine Based on an Attenuated CVB3 Vector and Evaluation of Immunological Responses in Mice, Vaccines. (2026) 14, no. 4, 10.3390/vaccines14040294, 294. 42042771 PMC13119997

[110] Kraan H. , Vrieling H. , Czerkinsky C. , Jiskoot W. , Kersten G. , and Amorij J.-P. , Buccal and Sublingual Vaccine Delivery, Journal of Controlled Release. (2014) 190, 580–592, 10.1016/j.jconrel.2014.05.060. 24911355 PMC7114675

[111] Kar S. , Devnath P. , Emran T. B. , Tallei T. E. , Mitra S. , and Dhama K. , Oral and Intranasal Vaccines Against SARS-CoV-2: Current Progress, Prospects, Advantages, and Challenges, Immunity, Inflammation and Disease. (2022) 10, no. 4, 10.1002/iid3.604. PMC8959423 35349752

[112] Davitt C. J. H. and Lavelle E. C. , Delivery Strategies to Enhance Oral Vaccination Against Enteric Infections, Advanced Drug Delivery Reviews. (2015) 91, 52–69, 10.1016/j.addr.2015.03.007. 25817337

[113] Rudin A. , Johansson E.-L. , Bergquist C. , and Holmgren J. , Differential Kinetics and Distribution of Antibodies in Serum and Nasal and Vaginal Secretions After Nasal and Oral Vaccination of Humans, Infection and Immunity. (1998) 66, no. 7, 3390–3396, 10.1128/IAI.66.7.3390-3396.1998. 9632610 PMC108357

[114] Xu H. , Cai L. , Hufnagel S. , and Cui Z. , Intranasal Vaccine: Factors to Consider in Research and Development, International Journal of Pharmaceutics. (2021) 609, 10.1016/j.ijpharm.2021.121180, 121180. 34637935

[115] van der Lubben I. M. , Verhoef J. C. , Borchard G. , and Junginger H. E. , Chitosan and Its Derivatives in Mucosal Drug and Vaccine Delivery, European Journal of Pharmaceutical Sciences. (2001) 14, no. 3, 201–207, 10.1016/S0928-0987(01)00172-5. 11576824

[116] Fujihashi K. , Sato S. , and Kiyono H. , Mucosal Adjuvants for Vaccines to Control Upper Respiratory Infections in the Elderly, Experimental Gerontology. (2014) 54, 21–26, 10.1016/j.exger.2014.01.006. 24440991 PMC3989367

[117] Gallichan W. S. , Woolstencroft R. N. , Guarasci T. , McCluskie M. J. , Davis H. L. , and Rosenthal K. L. , Intranasal Immunization With CpG Oligodeoxynucleotides as an Adjuvant Dramatically Increases IgA and Protection Against Herpes Simplex Virus-2 in the Genital Tract, The Journal of Immunology. (2001) 166, no. 5, 3451–3457, 10.4049/jimmunol.166.5.3451. 11207303

[118] McCluskie M. J. and Davis H. L. , CpG DNA Is a Potent Enhancer of Systemic and Mucosal Immune Responses Against Hepatitis B Surface Antigen With Intranasal Administration to Mice, The Journal of Immunology. (1998) 161, no. 9, 4463–4466, 10.4049/jimmunol.161.9.4463. 9794366

[119] Lycke N. , Recent Progress in Mucosal Vaccine Development: Potential and Limitations, Nature Reviews Immunology. (2012) 12, no. 8, 592–605, 10.1038/nri3251. 22828912

[120] Clements J. D. , Norton E. B. , and Papasian C. J. , The Mucosal Vaccine Adjuvant LT(R192G/L211A) or dmLT, mSphere. (2018) 3, no. 4, 10.1128/mSphere.00215-18. PMC6060342 30045966

[121] Lavelle E. C. and Ward R. W. , Mucosal Vaccines - Fortifying the Frontiers, Nature reviews. Immunology. (2021) 22, no. 4, 236–250, 10.1038/s41577-021-00583-2. PMC8312369 34312520

[122] Zhu Q. , Talton J. , and Zhang G. , et al.Large Intestine-Targeted, Nanoparticle-Releasing Oral Vaccine to Control Genitorectal Viral Infection, Nature Medicine. (2012) 18, no. 8, 1291–1296, 10.1038/nm.2866. PMC3475749 22797811

[123] Dong C. , Wang Y. , and Gonzalez G. X. , et al.Intranasal Vaccination With Influenza HA/GO-PEI Nanoparticles Provides Immune Protection Against Homo- and Heterologous Strains, Proceedings of the National Academy of Sciences. (2021) 118, no. 19, 10.1073/pnas.2024998118. PMC8126854 33941704

[124] Bal J. , Jung H.-Y. , Nguyen L. N. , Park J. , Jang Y.-S. , and Kim D.-H. , Evaluation of Cell-Surface Displayed Synthetic Consensus Dengue EDIII Cells as a Potent Oral Vaccine Candidate, Microbial Cell Factories. (2018) 17, no. 1, 10.1186/s12934-018-0994-8, 146. 30217208 PMC6138890

[125] Skwarczynski M. and Toth I. , Non-Invasive Mucosal Vaccine Delivery: Advantages, Challenges and the Future, Expert Opinion on Drug Delivery. (2020) 17, no. 4, 435–437, 10.1080/17425247.2020.1731468. 32059625

